# Clinical significance of urinary inflammatory biomarkers in patients with IgA nephropathy

**DOI:** 10.1186/s12882-024-03574-2

**Published:** 2024-04-22

**Authors:** Soo-Young Yoon, Jin Sug Kim, Su Woong Jung, Yang Gyun Kim, Ju-Young Moon, Sang-Ho Lee, Sung-Vin Yim, Hyeon Seok Hwang, Kyunghwan Jeong

**Affiliations:** 1 Division of Nephrology, Department of Internal Medicine, Kyung Hee University College of Medicine, Kyung Hee University Hospital, Seoul, Republic of Korea; 2 Division of Nephrology, Department of Internal Medicine, Kyung Hee University College of Medicine, Kyung Hee University Hospital at Gangdong, Seoul, Republic of Korea; 3 Department of Clinical Pharmacology, Kyung Hee University College of MedicineCenter, Seoul, Republic of Korea

**Keywords:** Biomarker, Cytokine, IgA nephropathy (IgAN), Progression, Urine

## Abstract

**Background:**

IgA nephropathy (IgAN) is the most common type of primary glomerulonephritis, although the definitive markers are unknown. We aimed to investigate the clinical significance of urinary cytokines in patients with IgAN.

**Methods:**

From 2009 to 2018, the patients were divided into three groups: IgAN (*n* = 191), disease control (*n* = 53), and normal control (*n* = 76). We used a multiplex enzyme-linked immunosorbent assay to measure 16 selected urinary inflammatory cytokines, evaluated the correlation between clinical and pathological features following regression analysis on progression.

**Results:**

The IgAN group exhibited significantly different levels of urinary cytokines compared to the normal control and disease control groups. Urinary levels of B-cell-activating factor, vascular endothelial growth factor receptor-2, monocyte chemoattractant protein-1, C–X–C motif chemokine 10, C–X–C motif ligand 16, epidermal growth factor (EGF), endocan, endostatin, growth/differentiation factor-15 (GDF-15), interleukin-6 (IL-6), mannose-binding lectin, transferrin receptor, and kidney injury molecule-1 were significantly correlated with both the estimated glomerular filtration rate and urine protein–creatinine ratio. In a multivariate Cox regression analysis, urinary EGF (hazard ratio [HR] 0.40, 95% confidence interval [CI] 0.17–0.95, *P* = 0.04), GDF-15 (HR 2.45, 95% CI 1.01–5.94, *P* = 0.048), and IL-6 (HR 3.02, 95% CI 1.05–8.64, *P* = 0.04) were associated with progression in IgAN.

**Conclusions:**

Urinary inflammatory biomarkers may serve as alternative predictive biomarkers in patients with IgAN. Further studies are needed to elucidate the physiological mechanisms and confirm the results.

**Supplementary Information:**

The online version contains supplementary material available at 10.1186/s12882-024-03574-2.

## Background

IgA nephropathy (IgAN) is the most common primary glomerulonephritis worldwide [1]. Its clinical course is variable and can progress to end-stage kidney disease (ESKD) in approximately 40% of patients within 20–30 years of diagnosis, necessitating dialysis or kidney transplant, both of which carry a substantial public health burden and result in high mortality [2]. In recent decades, considerable research has been conducted to identify biomarkers that predict the prognosis of IgAN. As constant inflammation could trigger the development and aggravation of IgAN, several experts on IgAN have focused on dysregulated inflammatory markers associated with a rapid decline in kidney function [3, 4].

Over 20 pre-clinical and clinical urinary biomarkers associated with IgAN have been identified, including monocyte chemoattractant protein-1 (MCP-1) and kidney injury molecule-1 (KIM-1) [5]. Despite these findings, an increase in urinary protein concentration is the only clinical urinary biomarker, including urine protein–creatinine ratio (uPCR) [5]. Studies continue to explore the relationship between urinary biomarkers and clinical features of patients with IgAN, and several biomarkers have been identified to detect risk factors and early disease progression in patients with IgAN [2, 6–8]. While kidney biopsy is the gold standard for diagnosing IgAN, novel urinary biomarkers may provide valuable prognostic information [7]. Therefore, integrating biomarkers with the existing prediction tools requires further effort.

The International IgAN Risk Prediction Tool (IIgANRPT) is the current strategy for predicting the high risk of chronic kidney disease (CKD) progression in IgAN [9]. This tool combines non-IgAN features, including estimated glomerular filtration rate (eGFR), mean arterial blood pressure, and amount of proteinuria and IgAN-related information from the diagnostic kidney biopsy, such as age at the biopsy date, Oxford classification score, renin–angiotensin system (RAS) blocker or immunosuppression usage, and race [9]. Urinary biomarkers with IIgANRPT may enhance the predictive value of IIgANRPT when used in combination.

This study aimed to verify the predictive value of urinary inflammatory biomarkers at the time of biopsy in patients with IgAN as well as that of noninvasive cytokine biomarkers, which are crucial for interpreting the clinical, histological, prognostic relevance of IgAN progression and identifying patients with IgAN suitable for immunomodulatory therapy.

## Methods

### Study design

Altogether, 320 individuals were enrolled in this study, not including acute kidney injury, autoimmune disorders, or cases of kidney transplant status. Of the patients, 191 patients from January 2008 to November 2018 were diagnosed with biopsy-proven IgAN; 53 patients with minimal change disease, membranous glomerulonephritis, and focal segmental glomerulosclerosis were considered disease controls; and 76 participants without kidney disease were included as healthy controls. Each patient’s renal function was assessed retrospectively one year after the biopsy, based on the diagnosis date, from January 2008 to November 2018. Clinical and laboratory data were collected at the time of renal biopsy to compare baseline characteristics among the IgAN, disease control, and healthy control groups including mean arterial blood pressure, age at the kidney biopsy, sex, RAS blocker usage, or history of immunosuppression therapy, and race. Urine samples were collected at the time of kidney biopsy and centrifuged at 2,000 ×*g* at 20 ℃ for 20 min. The pellets were separated from the supernatants and stored at − 80 °C in a deep freezer. The concentrations of the 16 selected urinary inflammatory biomarkers were measured for each patient according to the protocol outlined in the Methods section below.

### Measurement of clinical outcome

Function of the kidneys was evaluated by eGFR and calculated using the Chronic Kidney Disease Epidemiology Collaboration formula [10]. The level of proteinuria was measured using uPCR. All biopsy specimens were examined by the pathologists through standard methods. Regarding pathology, diagnostic assessment of IgAN was categorized according to the Oxford classification of IgAN from 2016 as follows: mesangial hypercellularity (M), endocapillary hypercellularity (E), segmental glomerulosclerosis (S), interstitial fibrosis/tubular atrophy (T), and crescentic lesions (C) [11].

The clinical outcome of the study was CKD progression, and disease progression in patients with IgAN was defined as a > 20% decrease in eGFR from baseline levels or in the condition of renal replacement therapy or kidney transplantation status after a one-year follow-up. Progressors or non-progressors were defined as those who experienced a predefined decline in kidney function.

### Selection and measurements of urinary inflammatory markers by multiplex enzyme-linked immunosorbent assay (ELISA)

Representative online databases, Medline, and Scopus, were searched using the following terms: ((immunoglobulin A nephropathy) OR (IgAN)) AND (((((((nephritis) OR (renal injury*)) OR (kidney injury*)) OR (renal damage*)) OR (kidney damage)) OR (ckd)) OR (chronic kidney disease))) AND (urine*)) AND (biomarker*). Twenty-four reviews, meta-analyses, randomized control trials, and cohort studies that were accessible with at least one English abstract were selected. Several urinary biomarkers have been suggested to evaluate IgAN progression. We selected the following 16 candidate urinary inflammatory biomarkers: B-cell-activating factor (BAFF), vascular endothelial growth factor receptor-2 (VEGFR2), MCP-1, regulated on activation, normal T cell expressed and secreted (RANTES), C–X–C motif ligand 10 (CXCL10), C–X–C motif ligand 16 (CXCL16), epidermal growth factor (EGF), endocan, endostatin, growth/differentiation factor-15 (GDF-15), interferon γ (IFNγ), interleukin-6 (IL-6), mannose-binding lectin (MBL), nephrin, transferrin receptor (TfR), and KIM-1 [3, 12–25].

Each urinary cytokine or chemokine was measured by multiplex ELISA using a customized Magnetic Luminex Screening Assay, according to the manufacturer’s protocols (R&D Systems, Minneapolis, MN, USA). Concentration of each inflammatory biomarker using the Luminex 200 (Luminex, Austin, TX, USA) was calculated according to the standard curves of the reference sample. The levels of these biomarkers were normalized for urine creatinine concentration (ng/gCr) following log_10_ transformation of normalized measurements to account for any influence that urinary dilution or concentration based on a patient’s hydration status may have on biomarker levels.

### Statistical analyses

Baseline characteristics of the study population are represented as mean ± standard deviation or number of subjects with percentage. The levels of urinary inflammatory biomarkers were normally distributed after log_10_ transformation. We performed the Kruskal–Wallis and Mann–Whitney tests to compare continuous variables, while categorical variables between groups were analyzed using the Pearson χ^2^-test. Mean ± standard error were represented by error bars in figures. When comparing among three groups, Bonferroni correction was applied to the *p* values. In Spearman’s correlation analyses, urinary cytokine levels were compared with eGFR and uPCR. Before conducting the multivariate analysis, we checked for multicollinearity by ensuring that the variance inflation factor for each adjustment variable was less than 8. After confirming the absence of multicollinearity, we performed multivariate analysis for each biomarker while including the confirmed adjustment variables. Previous research has demonstrated that the biomarkers in our study interact with each other [15, 26–28]. Therefore, we have chosen to analyze each biomarker individually rather than using a multivariate regression analysis. Multivariate Cox proportional hazard regression model was presented as hazard ratios (HRs) and 95% confidence intervals (CIs) for each urinary inflammatory biomarkers. Data were adjusted for age, sex, hypertension, eGFR, and uPCR. Receiver operating characteristic (ROC) curves and areas under the ROC curve (AUC) represented the clinical value of urinary inflammatory biomarkers in patients with IgAN. All the statistical analyses and graphs were represented via SPSS for Windows (version 22.0; IBM, Armonk, NY, USA) and GraphPad Prism 8.0. Significance was set at *p* < 0.05 for all the tests. Additionally, internal validation analyses for 1,000 times with 90% of the data were conducted using R software version 4.1.3 (R Foundation for Statistical Computing).

## Results

### Baseline clinical characteristics of the study population

The baseline demographics of the enrolled patients are presented in Table 1. The median age was 41.5 ± 15.9 years, and 47.2% of the patients were male. The IgAN group had significantly lower eGFR levels than the normal control group (83.0 ± 34.5 versus 109.6 ± 15.6 mL/min/1.73 m^2^, *P* < 0.05). uPCR level was significantly different among the IgAN group, disease control group, and healthy control group (1.7 ± 2.1 versus 5.4 ± 5.6 versus 0.1 ± 0.1 g/g, respectively; *P* < 0.001).

**Table 1:** Baseline characteristics of the study population

	IgAN (*n* = 191)	Disease control (*n* = 53)	Normal control (*n* = 76)	P^d^
Age, years	41.3 ± 15.3	46.4 ± 17.7^b^	38.4 ± 15.4	0.018
Male, n (%)	99 (51.8)^a^	33 (62.3)^b^	19 (25.0)^c^	< 0.0001
HTN, n (%)	80 (41.9)	20 (37.7)	0 (0.0)^c^	< 0.0001
DM, n (%)	10 (5.2)^a^	10 (18.9)^b^	0 (0.0)^c^	< 0.0001
BMI, kg/m^2^	23.9 ± 3.3	24.8 ± 3.2	22.9 ± 3.5^c^	0.005
Albumin, g/dL	3.9 ± 0.6^a^	2.9 ± 0.9^b^	4.5 ± 0.2^c^	< 0.0001
Creatinine, mg/dL	1.2 ± 0.9^a^	0.9 ± 0.6^b^	0.7 ± 0.1^c^	< 0.0001
eGFR, ml/min/1.73 m^2^	83.0 ± 34.5^a^	96.7 ± 33.6^b^	109.6 ± 15.6^c^	< 0.0001
C3, mg/dL	108.9 ± 20.7	116.6 ± 20.5	N/A	0.015
IgA, mg/dL	305.5 ± 103.9	242.5 ± 87.4	N/A	< 0.0001
Urine PCR, g/gCr	1.7 ± 2.1^a^	5.4 ± 5.6^b^	0.1 ± 0.1^c^	< 0.0001

BMI, body mass index; DM, diabetes mellitus; eGFR, estimated glomerular filtration rate; HPF, high-power field; HTN, hypertension; IgAN, IgA nephropathy; PCR, protein–creatinine ratio; RBC, red blood cell

Different alphabets are significant differences between groups in the results of the Bonferroni post hoc analysis

^a^*P* < 0.05, the other two respective groups vs. IgAN

^b^*P* < 0.05, the other two respective groups vs. disease control

^c^*P* < 0.05, the other two respective groups vs. normal control

^d^*P* < 0.017 (0.05 divided by 3) was considered as significant difference

**Fig. 1:**
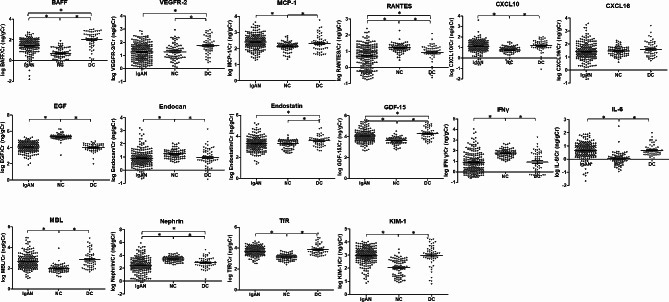
Levels of urinary inflammatory biomarkers according to IgAN, disease control, and normal control group BAFF, B-cell-activating factor; CXCL10, C–X–C motif chemokine 10; CXCL16, C–X–C motif ligand 16; EGF, epidermal growth factor; GDF-15, growth/differentiation factor-15; IFNγ, interferon γ; IL-6, interleukin-6; KIM-1, kidney injury molecule-1; MBL, mannose-binding lectin; MCP-1, monocyte chemoattractant protein-1; RANTES, regulated on activation, normal T cell expressed and secreted; TfR, transferrin receptor; VEGFR-2, vascular endothelial growth factor receptor-2 *P < 0.05 Multiple comparisons were performed using the Mann-Whitney test for each of the 16 urinary inflammatory biomarkers between each of the groups

Figure 1 shows that the IgAN group had higher levels of eight urinary inflammatory biomarkers compared to the normal control group: BAFF (1.42 ± 0.70 versus 0.68 ± 0.56 ng/gCr), MCP-1 (2.41 ± 0.45 versus 2.13 ± 0.28 ng/gCr), CXCL10 (1.13 ± 0.52 versus 0.75 ± 0.43 ng/gCr), GDF-15 (4.07 ± 0.54 versus 3.62 ± 0.33 ng/gCr), IL-6 (0.66 ± 0.58 versus 0.04 ± 0.47 ng/gCr), MBL (2.65 ± 0.69 versus 1.98 ± 0.36 ng/gCr), TfR (3.71 ± 0.38 versus 3.16 ± 0.30 ng/gCr), and KIM-1 (2.96 ± 0.61 versus 2.06 ± 0.64 ng/gCr). In contrast, the IgAN group exhibited lower levels of five urinary inflammatory biomarkers compared to the normal control group: RANTES (0.68 ± 0.69 versus 1.20 ± 0.27 ng/gCr), EGF (4.07 ± 0.57 versus 5.32 ± 0.37 ng/gCr), endocan (0.90 ± 0.63 versus 1.24 ± 0.35 ng/gCr), IFNγ (0.91 ± 0.92 versus 1.77 ± 0.41 ng/gCr), and nephrin (2.44 ± 1.20 versus 3.44 ± 0.43 ng/gCr). Additionally, the IgAN group showed significantly lower levels of BAFF, VEGFR-2, RANTES, endostatin, GDF-15, and nephrin compared to the disease control group.

### Urinary cytokine biomarkers and clinicopathological correlations in patients with IgAN

**Fig. 2:**
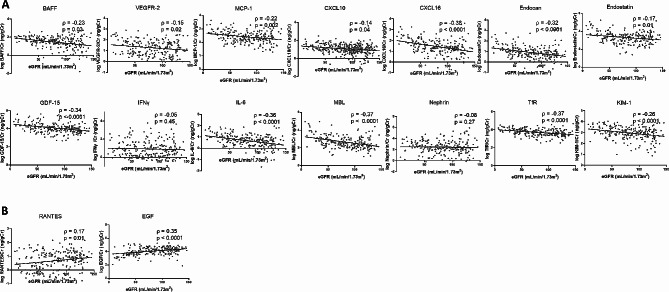
Correlation between urinary inflammatory biomarker levels and estimated glomerular filtration rate in the patients with IgAN. **A** Negative correlation. **B** Positive correlation BAFF, B-cell-activating factor; CXCL10, C–X–C motif chemokine 10; CXCL16, C–X–C motif ligand 16; EGF, epidermal growth factor; eGFR, estimated glomerular filtration rate; GDF-15, growth/differentiation factor-15; IFNγ, interferon γ; IL-6, interleukin-6; KIM-1, kidney injury molecule-1; MBL, mannose-binding lectin; MCP-1, monocyte chemoattractant protein-1; RANTES, regulated on activation, normal T cell expressed and secreted; TfR, transferrin receptor; VEGFR-2, vascular endothelial growth factor receptor-2 Spearman’s rank correlation coefficient test was used for the statistical analysis

**Fig. 3:**
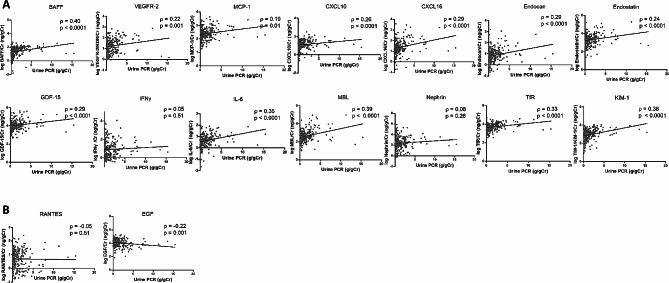
Correlation between urinary inflammatory biomarker levels and urine protein–creatinine ratio in the patients with IgAN. **A** Positive correlation. **B** Negative correlation BAFF, B-cell-activating factor; CXCL10, C–X–C motif chemokine 10; CXCL16, C–X–C motif ligand 16; EGF, epidermal growth factor; eGFR, estimated glomerular filtration rate; GDF-15, growth/differentiation factor-15; IFNγ, interferon γ; IL-6, interleukin-6; KIM-1, kidney injury molecule-1; MBL, mannose-binding lectin; MCP-1, monocyte chemoattractant protein-1; PCR, protein–creatinine ratio; RANTES, regulated on activation, normal T cell expressed and secreted; TfR, transferrin receptor; VEGFR-2, vascular endothelial growth factor receptor-2 Spearman’s rank correlation coefficient test was used for the statistical analysis

As presented in Fig. 2A, eGFR was negatively correlated with urinary BAFF (ρ = -0.23), VEGFR-2 (ρ = -0.15), MCP-1 (ρ = -0.22), CXCL10 (ρ = -0.14), CXCL16 (ρ = -0.38), endocan (ρ = -0.32), endostatin (ρ = -0.17), GDF-15 (ρ = -0.34), IL-6 (ρ = -0.36), MBL (ρ = -0.37), TfR (ρ = -0.37), and KIM-1 (ρ = -0.26) with *P* < 0.05 for all the aforementioned 12 biomarkers in the patients with IgAN. RANTES (ρ = 0.17, *P* = 0.01) and EGF (ρ = 0.35, *P* < 0.001) levels were positively correlated with eGFR (Fig. 2B) By contrast, uPCR was positively correlated with urinary BAFF (ρ = 0.40), VEGFR-2 (ρ = 0.22), MCP-1 (ρ = 0.19), CXCL10 (ρ = 0.26), CXCL16 (ρ = 0.29), endocan (ρ = 0.29), endostatin (ρ = 0.24), GDF-15 (ρ = 0.29), IL-6 (ρ = 0.35), MBL (ρ = 0.39), TfR (ρ = 0.33), and KIM-1 (ρ = 0.36), with *P* < 0.05 for the 12 biomarkers (Fig. 3A). uPCR demonstrated an insignificant trend with RANTES levels (ρ = -0.05, *P* = 0.51), whereas uPCR demonstrated a negative trend toward significance with EGF levels (ρ = -0.22, *P* = 0.001) (Fig. 3B).

Increases in CXCL16, IFNγ, and TfR levels were significant in patients with mesangial hypercellularity (M0 versus M1) (Table S1). Urinary CXCL10 levels were significantly higher in patients with greater endocapillary hypercellularity (E0 versus E1) according to the Oxford classification (Table S1). Three urinary levels of CXCL10, nephrin, and KIM-1 were elevated in patients with segmental glomerulosclerosis (S0 versus S1). Additionally, four urinary levels of CXCL16, endocan, MBL, and TfR were elevated in patients with interstitial fibrosis/tubular atrophy (T0 versus T1–2). Finally, urinary TfR levels were significantly different in patients with the extent of crescent lesions (C0 versus C1–2) according to the Oxford classification.

### Urinary inflammatory biomarkers for predicting disease progression in patients with IgAN

Twenty-one (11.0%) patients met the criteria for disease progression, defined as a > 20% decline in eGFR or progression to ESKD within one-year of follow-up (Table 2). The progressors were older (*P* = 0.001) and had a higher prevalence of hypertension (*P* < 0.001), lower albumin levels (*P* = 0.01), lower eGFR levels (*P* < 0.001), and higher uPCR levels (*P* < 0.001) than the non-progressors. The use of RAS blockers, angiotensin-converting enzyme inhibitors, and immunosuppressants was not significantly different between the progressors and non-progressors.

**Table 2:** Clinical characteristics of the patients with IgAN according to disease progression

	Progression (*n* = 21)	Non-progression (*n* = 170)	*P*
Age, years	53.3 ± 15.4	40.0 ± 14.4	0.001
Male, n (%)	11 (52.4)	74 (43.5)	0.44
HTN, n (%)	16 (76.2)	61 (35.9)	< 0.001
DM, n (%)	2 (9.5)	9 (5.3)	0.43
BMI, kg/m^2^	24.3 ± 3.3	23.9 ± 3.3	0.42
Albumin, g/dL	3.7 ± 0.4	3.9 ± 0.6	0.01
IgA, mg/dL	336.6 ± 88.5	304.5 ± 104.9	0.07
eGFR, ml/min/1.73 m^2^	55.9 ± 34.9	86.9 ± 32.7	< 0.001
Urine PCR, g/gCr	2.7 ± 2.3	1.4 ± 1.8	< 0.001
Use of ARB or ACEi, n (%)	16 (76.2)	124 (72.9)	0.75
Use of immunosuppressant, n (%)	12 (57.1)	101 (59.4)	0.84
Oxford classification M			0.76
0	11 (52.4)	95 (55.9)	
1	10 (47.6)	75 (44.1)	
Oxford classification E			0.68
0	16 (76.2)	136 (80.0)	
1	5 (76.9)	34 (20.0)	
Oxford classification S			0.80
0	13 (61.9)	110 (64.7)	
1	8 (38.1)	60 (35.3)	
Oxford classification T			0.96
0	18 (85.7)	145 (85.3)	
1, 2	3 (14.3)	25 (14.7)	
Oxford classification C			0.92
0	17 (81.0)	145 (85.3)	
1, 2	4 (9.0)	24 (14.7)	

ARB, angiotensin II receptor blockers; ACEi, angiotensin II converting enzyme inhibitors; BMI, body mass index; C, crescents; DM, diabetes mellitus; E, endocapillary hypercellularity; eGFR, estimated glomerular filtration rate; HPF, high-power field; HTN, hypertension; M, mesangial hypercellularity; PCR, protein–creatinine ratio; RBC, red blood cell; S, segmental glomerulosclerosis; T, interstitial fibrosis/tubular atrophy

In multivariate Cox regression analysis after the one-year follow-up, cytokine biomarkers (GDF-15 [HR 2.45, 95% CI 1.01–5.94, *P* = 0.048], IL-6 [HR 3.02, 95% CI 1.05–8.64, *P* = 0.04], and EGF [HR 0.40, 95% CI 0.17–0.95, *P* = 0.04]) were significantly associated with CKD progression in the patients with IgAN in Table 3. In addition, internal validation analyses for 1,000 times with 90% of the data showed statistical significance for IL-6 in Table S2. GDF-15 tended to increase as a predictive marker of disease progression.

**Table 3:** Predictors of disease progression in the multivariate Cox regression analysis

	Multivariate analysis	
Variables	HR (95% CI) ^b^	*P*
BAFF^a^	1.94 (0.59–6.39)	0.28
VEGFR-2^a^	1.63 (0.70–3.77)	0.26
MCP-1^a^	1.88 (0.57–6.16)	0.30
RANTES^a^	0.99 (0.52–1.88)	0.97
CXCL10^a^	1.32 (0.44–3.96)	0.63
CXCL16^a^	1.61 (0.69–3.71)	0.27
EGF^a^	0.40 (0.17–0.95)	0.04
Endocan^a^	1.60 (0.68–3.75)	0.28
Endostatin^a^	1.37 (0.61–3.05)	0.45
GDF-15^a^	2.45 (1.01–5.94)	0.048
IFNγ^a^	0.91 (0.53–1.58)	0.75
IL-6^a^	3.02 (1.05–8.64)	0.04
MBL^a^	1.92 (0.89–4.13)	0.10
Nephrin^a^	1.03 (0.66–1.62)	0.89
TfR^a^	0.66 (0.17–2.55)	0.55
KIM-1^a^	1.85 (0.69–4.93)	0.22

BAFF, B-cell-activating factor; CI, confidence interval; CXCL10, C–X–C motif chemokine 10; CXCL16, C–X–C motif ligand 16; DM, diabetes mellitus; HTN, hypertension; EGF, epidermal growth factor; eGFR, estimated glomerular filtration rate; GDF-15, growth/differentiation factor-15; HR, hazard ratio; IFNγ, interferon γ; IL-6, interleukin-6; KIM-1, kidney injury molecule-1; MBL, mannose-binding lectin; MCP-1, monocyte chemoattractant protein-1; PCR, protein–creatinine ratio; RANTES, regulated on activation, normal T cell expressed and secreted; TfR, transferrin receptor; VEGFR-2, vascular endothelial growth factor receptor-2

^a^Biomarker values are expressed relative to urine creatinine concentration and then log-transformed

^b^Multivariate analysis was conducted by adjusting for age, sex, HTN, eGFR, and PCR for each biomarker separately

**Fig. 4:**
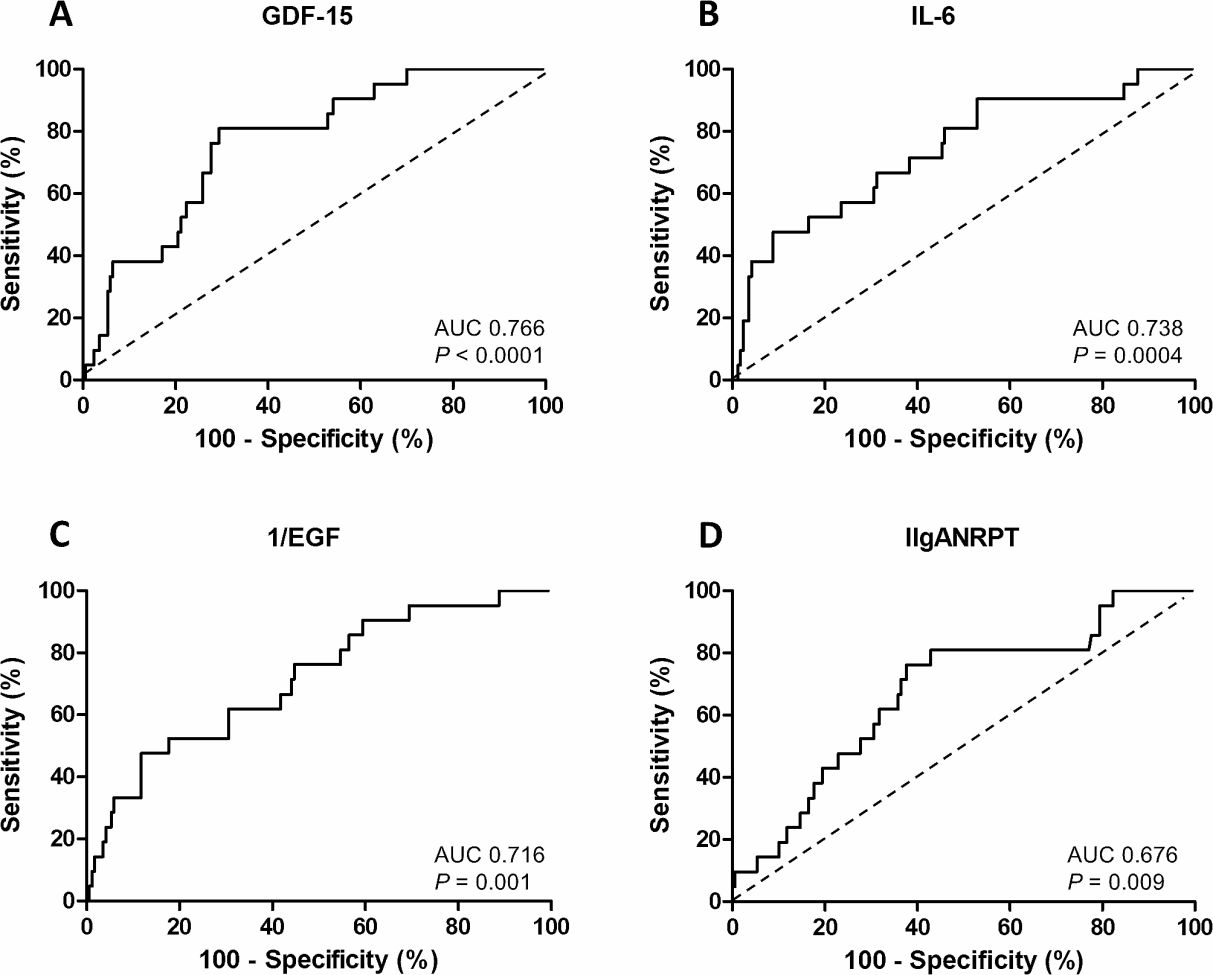
Receiver operating characteristic curve analysis of urinary biomarkers in patients with IgAN. **A** growth/differentiation factor-15 (GDF-15). **B** interleukin-6 (IL-6). **C** reverse of epidermal growth factor (EGF). **D** International IgA nephropathy Risk Prediction Tool (IIgANRPT)

To evaluate the discriminatory values for disease progression, an ROC curve was constructed for the patients with IgAN. As predictive factors after the one-year follow up, urinary GDF-15 (AUC 0.77, 95% CI 0.67–0.86, *P* < 0.001), IL-6 (AUC 0.74, 95% CI 0.62–0.86, *P* < 0.001), and reverse of EGF (AUC 0.72, 95% CI 0.60–0.83, *P* = 0.001) were as effective as IIgANRPT per se in assessing IgAN progression (AUC 0.68, 95% CI, 0.56–0.79; *P* = 0.01) (Fig. 4).

## Discussion

Although further research is ongoing, the pathogenesis of inflammatory biomarkers and IgAN is still unclear. Studies using urine samples may be useful due to their non-invasive nature. Accordingly, we aimed to measure the levels of 16 urinary inflammatory biomarkers, selected based on literature review, at the time of biopsy to explore their association with disease progression. Comparing the levels of each biomarker between the IgAN group and the normal control group, we found that eight urinary cytokines showed higher levels in the normal control group, while five urinary cytokines showed lower levels. Therefore, most of the urinary inflammatory biomarkers showed significant differences between the disease group and the normal control group. In addition, the levels of 14 urinary inflammatory biomarkers tended to increase with decreasing eGFR and increase with increasing uPCR. Conversely, the remaining 2 biomarkers tended to increase with increasing eGFR and increased with decreasing uPCR. Finally, among the 16 urinary inflammatory biomarkers we studied, GDF-15, IL-6, and the inverse of EGF were most likely to be associated with disease progression. Such non-invasive urinary biomarkers may be as predictive of disease progression as IIgANRPT.

The GDF-15 levels were higher in patients with IgAN, and elevation of GDF-15 may be involved in the decline of kidney function. GDF-15 is a member of the transforming growth factor-β (TGF-β) cytokine superfamily, and its level of plasma GDF-15 increases in response to ischemic tissue damage and other proinflammatory cytokines [29]. In previous studies, plasma GDF-15 levels were associated with renal function and development of interstitial fibrosis and tubular atrophy in patients with IgAN [23, 30]. Perez-Gomez et al. have reported that urinary GDF-15 levels were also associated with kidney histological reports, mortality, and progression to ESKD [31]. Specifically, our study is the first study to demonstrate the predictive value of urinary GDF-15 for disease progression in patients with IgAN.

The urinary IL-6 expression was greater in patients with IgAN, negatively correlated with eGFR, and positively correlated with uPCR, indicating the association of IL-6 with kidney disease progression. IL-6 induces the Janus kinase/signal transducer and activator of transcription 3 pathway, which can produce galactose-deficient IgA1, a critical molecule in IgAN pathogenesis [32, 33]. In previous follow-up studies, patients with IgAN also showed elevated urinary IL-6, and elevated urinary level of IL-6 was significantly associated with CKD aggravation [6, 8, 15]. Hence, further studies are needed to elucidate the association between IgAN progression and IL-6 overexpression in urine.

Urinary EGF levels may serve as a noninvasive biomarker for the extent of interstitial fibrosis, and this result was also consistent with a decreasing trend in urinary levels of EGF in patients with a higher score (1 or 2) of T-lesion in the Oxford classification for patients with IgAN [15, 24]. Several previous studies have suggested that urinary excretion of EGF is mainly produced by the ascending portion of Henle’s loop or distal convoluted tubule, modulates the kidney tissue response to tubulointerstitial injury, and accelerates recovery from acute tubular injury in animal models [34]. In human-based studies, reduced urine EGF levels have been observed in patients with IgAN, and urine EGF demonstrated an inverse correlation with the severity of tubulointerstitial damage or T-lesions of the Oxford classification for patients with IgAN [15, 35, 36]. These findings tended to be consistent with our study results, although they did not reach statistical significance. Lack of urinary EGF expression may play a major role in aggravating tubulointerstitial injury in patients with IgAN. Furthermore, the three urinary inflammatory biomarkers demonstrated better discriminative prediction of clinical outcomes in patients with IgAN than conventional IIgANRPT in the ROC analysis, and these more powerful candidates need more validation.

The strength of this study is that the urinary inflammatory biomarkers were selected through analyzing evidence-based articles. The results of this study are meaningful in that they analyzed the correlations between selected urinary biomarkers and clinicopathological characteristics of patients with IgAN. We also compared each predictive value of one-year clinical outcome between selected biomarkers.

Nevertheless, this study had several limitations. First, the number of participants was relatively not large. Therefore, these results need to be validated in a large-scale cohort. Second, urinary cytokine levels were assessed only once at the time of kidney biopsy, which could lead to an incorrect correlation due to a one-time checkup. Conducting repeated long-term follow-up studies, rather than a single analysis, to further analyze changes in urinary inflammatory biomarkers could yield more clinically meaningful outcomes. Third, different treatments could affect kidney function decline in patients with IgAN, and patients with IgAN in this study were prescribed RAS blockers or immunosuppressants when they met the criteria for treatment guidelines. Fourth, it could be argued that identified inflammatory biomarkers are also able to be detected in CKD patients with other diseases. Finally, a one-year follow-up period may be insufficient for assessing CKD progression, considering the long prognosis of IgAN. Disease progression, defined as a decline in eGFR by 20%, may have led to the under- or overestimation of worsening clinical outcomes in this study.

## Conclusions

The urinary inflammatory biomarkers in the IgAN group were dysregulated compared with those in the disease control and healthy control groups. Specific urinary cytokines, such as GDF-15, IL-6, and EGF, significantly strengthened the predictability of IgAN prognosis. These candidates, as early prognostic biomarker candidates, may assist in the formulation of therapeutic strategies for patients with IgAN. Further studies are needed to validate these noninvasive urinary inflammatory biomarkers as clinical indicators of kidney damage in patients with IgAN.

### Electronic supplementary material

Below is the link to the electronic supplementary material.

Supplementary Material 1

## Data Availability

The authors of this study declare that all main data within the paper are available. All other data are available upon reasonable request to the corresponding authors.

## Abbreviations

IgAN: IgA nephropathy
ESKD: end-stage kidney disease
MCP-1: monocyte chemoattractant protein-1
KIM-1: kidney injury molecule-1
uPCR: urine protein–creatinine ratio
IIgANRPT: International IgAN Risk Prediction Tool
CKD: chronic kidney disease
eGFR: estimated glomerular filtration rate
RAS: renin–angiotensin system
M: mesangial hypercellularity
E: endocapillary hypercellularity
S: segmental glomerulosclerosis
T: interstitial fibrosis/tubular atrophy
C: crescentic lesions
BAFF: B-cell-activating factor
VEGFR2: vascular endothelial growth factor receptor-2
RANTES: regulated on activation, normal T cell expressed and secreted
CXCL10: C–X–C motif ligand 10
CXCL16: C–X–C motif ligand 16
EGF: epidermal growth factor
GDF-15: growth/differentiation factor-15
IFNγ: interferon γ
IL-6: interleukin-6
MBL: mannose-binding lectin
TfR: transferrin receptor
HR: hazard ratio
ROC: Receiver operating characteristic
AUC: areas under the ROC curve
