# Supplementary material for: Clinical significance of urinary inflammatory biomarkers in patients with IgA nephropathy
Source: BMC Nephrol. 2024 Apr 22;25:142. doi: 10.1186/s12882-024-03574-2 (PMC11036669; doi:10.1186/s12882-024-03574-2)
Supplement: Supplementary file 1 — Supplementary Material 1 [file 12882_2024_3574_MOESM1_ESM.docx]

**Table S1**. Urinary inflammatory biomarker levels according to pathologic findings based on the Oxford classification (MEST-C).

| Oxford classification | M | | | E | | | S | | | T | | C | | | |  |
| --- | --- | --- | --- | --- | --- | --- | --- | --- | --- | --- | --- | --- | --- | --- | --- | --- |
| Score | 0 | 1 | *P* | 0 | 1 | *P* | 0 | 1 | *P* | 0 | 1,2 | *P* | 0 | 1,2 | *P* | |
| n (%) | 106  (55.5) | 85  (44.5) |  | 152  (79.6) | 39  (20.4) |  | 123  (64.4) | 68  (35.6) |  | 163  (85.3) | 28  (14.7) |  | 164  (85.9) | 27  (14.1) |  | |
| BAFF^a^ | 1.36 ± 0.77 | 1.51 ± 0.70 | 0.14 | 1.39 ± 0.70 | 1.53± 0.71 | 0.13 | 1.45 ± 0.65 | 1.37± 0.78 | 0.76 | 1.39 ± 0.73 | 1.58 ± 0.46 | 0.15 | 1.41 ± 0.72 | 1.51 ± 0.62 | 0.41 | |
| VEGFR-2^a^ | 1.21± 0.68 | 1.33 ± 0.64 | 0.15 | 1.23 ± 0.67 | 1.35 ± 0.64 | 0.30 | 1.34 ± 0.64 | 1.12 ± 0.69 | 0.01 | 1.24± 0.68 | 1.40 ± 0.56 | 0.19 | 1.25± 0.67 | 1.28 ± 0.65 | 0.91 | |
| MCP-1^a^ | 2.39 ± 0.46 | 2.43 ± 0.43 | 0.86 | 2.39 ± 0.45 | 2.47 ± 0.42 | 0.32 | 2.40 ± 0.44 | 2.42 ± 0.45 | 0.64 | 2.41 ± 0.45 | 2.42 ± 0.40 | 0.83 | 2.40 ± 0.45 | 2.47 ± 0.42 | 0.39 | |
| RANTES^a^ | 0.73 ± 0.67 | 0.62 ± 0.70 | 0.13 | 0.72 ± 0.64 | 0.58 ± 0.81 | 0.44 | 0.67 ± 0.66 | 0.70 ± 0.74 | 0.40 | 0.72 ± 0.68 | 0.47 ± 0.69 | 0.06 | 0.71 ± 0.68 | 0.57 ± 0.70 | 0.37 | |
| CXCL10^a^ | 1.13 ± 0.47 | 1.12 ± 0.57 | 0.57 | **1.09 ± 0.47** | **1.25 ± 0.65** | **0.03** | 1.08 ± 0.56 | **1.21 ± 0.41** | **0.04** | 1.12 ± 0.52 | 1.16 ± 0.53 | 0.88 | 1.11 ± 0.53 | 1.22 ± 0.45 | 0.28 | |
| CXCL16^a^ | **1.32 ± 0.74** | **1.53 ± 0.63** | **0.03** | 1.40 ± 0.71 | 1.44 ± 0.67 | 0.76 | 1.43 ± 0.70 | 1.38 ± 0.70 | 0.89 | **1.36 ± 0.71** | **1.70 ± 0.56** | **0.003** | 1.38 ± 0.70 | 1.53 ± 0.70 | 0.10 | |
| EGF^a^ | 4.10 ± 0.54 | 4.03 ± 0.61 | 0.40 | 4.04 ± 0.60 | 4.16 ± 0.48 | 0.33 | 4.09 ± 0.54 | 4.04 ± 0.62 | 0.87 | 4.10 ± 0.56 | 3.91 ± 0.61 | 0.053 | 4.08 ± 0.57 | 4.03 ± 0.56 | 0.48 | |
| Endocan^a^ | 0.91 ± 0.67 | 0.89 ± 0.58 | 0.61 | 0.87 ± 0.64 | 1.00 ± 0.61 | 0.29 | 0.88 ± 0.67 | 0.95 ± 0.56 | 0.31 | 0.86 ± 0.64 | **1.11 ± 0.54** | **0.005** | 0.88 ± 0.63 | 1.00 ± 0.64 | 0.12 | |
| Endostatin^a^ | 3.29 ± 0.66 | 3.35 ± 0.62 | 0.47 | 3.30 ± 0.66 | 3.37 ± 0.62 | 0.55 | 3.33 ± 0.62 | 3.29 ± 0.70 | 0.75 | 3.29 ± 0.66 | 3.45 ± 0.56 | 0.08 | 3.30 ± 0.65 | 3.35 ± 0.63 | 0.40 | |
| GDF-15^a^ | 4.02 ± 0.51 | 4.14 ± 0.58 | 0.27 | 4.04 ± 0.53 | 4.16 ± 0.58 | 0.31 | 4.08 ± 0.53 | 4.04 ± 0.57 | 0.92 | 4.06 ± 0.56 | 4.12 ± 0.42 | 0.29 | 4.07 ± 0.56 | 4.07 ± 0.49 | 0.57 | |
| IFNγ^a^ | **0.80 ± 0.91** | **1.05 ± 0.92** | **0.03** | 0.89 ± 0.94 | 0.98 ± 0.84 | 0.36 | 0.99 ± 0.99 | 0.75 ± 0.75 | 0.23 | 0.89 ± 0.92 | 1.00 ± 0.91 | 0.41 | 0.90 ± 0.92 | 0.97 ± 0.97 | 0.75 | |
| IL-6^a^ | 0.60 ± 0.59 | 0.74 ± 0.55 | 0.18 | 0.65 ± 0.58 | 0.70 ± 0.57 | 0.51 | 0.62 ± 0.62 | 0.74 ± 0.49 | 0.12 | 0.64 ± 0.61 | 0.78 ± 0.38 | 0.19 | 0.65 ± 0.58 | 0.74 ± 0.58 | 0.17 | |
| MBL^a^ | 2.62 ± 0.64 | 2.68 ± 0.75 | 0.92 | 2.63 ± 0.68 | 2.71 ± 0.71 | 0.32 | 2.62 ± 0.65 | 2.69 ± 0.76 | 0.84 | 2.61 ± 0.68 | **2.86 ± 0.70** | **0.050** | 2.62 ± 0.69 | 2.80 ± 0.70 | 0.20 | |
| Nephrin^a^ | 2.41 ± 1.25 | 2.48 ± 1.12 | 0.35 | 2.39 ± 1.25 | 2.60 ± 1.01 | 0.16 | 2.58 ± 1.21 | 2.16 ± 1.13 | 0.03 | 2.43 ± 1.23 | 2.44 ± 0.99 | 0.72 | 2.46 ± 1.21 | 2.29 ± 1.15 | 0.77 | |
| TfR^a^ | **3.65 ± 0.40** | **3.78 ± 0.35** | **0.01** | 3.68 ± 0.39 | 3.80 ± 0.34 | 0.09 | 3.69 ± 0.38 | 3.74 ± 0.39 | 0.44 | 3.68 ± 0.38 | **3.84 ± 0.37** | **0.02** | 3.68 ± 0.37 | **3.85 ± 0.44** | **0.03** | |
| KIM-1^a^ | 2.95 ± 0.59 | 2.96 ± 0.63 | 0.75 | 2.92 ± 0.60 | 3.06 ± 0.64 | 0.15 | 2.84 ± 0.61 | **3.17 ± 0.54** | **<0.001** | 2.94 ± 0.61 | 3.05 ± 0.58 | 0.37 | 2.93 ± 0.61 | 3.11 ± 0.57 | 0.19 | |

BAFF, B-cell-activating factor; C, crescents; CXCL10, C–X–C motif chemokine 10; CXCL16, C–X–C motif ligand 16; E, endocapillary hypercellularity; EGF, epidermal growth factor; GDF-15, growth/differentiation factor-15; IFNγ, interferon γ; IL-6, interleukin-6; KIM-1, kidney injury molecule-1; MBL, mannose-binding lectin; M, mesangial hypercellularity; MCP-1, monocyte chemoattractant protein-1; RANTES, regulated on activation, normal T cell expressed and secreted; S, segmental glomerulosclerosis; T, interstitial fibrosis/tubular atrophy; TfR, transferrin receptor; VEGFR-2, vascular endothelial growth factor receptor-2.

^a^Biomarker values are expressed relative to urine creatinine concentration and then log-transformed.

The numbers in bold indicate a significant difference (*p*<0.05).

**Table S2**. Validation for predictors of disease progression in the multivariate Cox regression analysis.

|  | Multivariate analysis | |
| --- | --- | --- |
| Variables | HR (95% CI) ^b,c^ | *P* |
| EGF^a^ | 0.45 (0.13-1.62) | 0.24 |
| GDF-15^a^ | 4.38 (0.96-2.01) | 0.09 |
| IL-6^a^ | 9.72 (2.29-4.26) | 0.01 |

CI, conﬁdence interval; HTN, hypertension; EGF, epidermal growth factor; eGFR, estimated glomerular filtration rate; GDF-15, growth/differentiation factor-15; HR, hazard ratio; IL-6, interleukin-6; PCR, protein–creatinine ratio.

^a^Biomarker values are expressed relative to urine creatinine concentration and then log-transformed.

^b^Multivariate analysis was conducted by adjusting for age, sex, HTN, eGFR, and PCR for each biomarker separately.

^c^For internal validation, the mean HR with CI was calculated after Cox regression analysis for 1,000 times with 90% of the data for each biomarker separately.
